# Syringocystadenocarcinoma papilliferum – ein Fallbericht

**DOI:** 10.1007/s00105-024-05437-1

**Published:** 2024-12-05

**Authors:** Deborah Winkler, Christoforus Christofi, Uta Bachter, Karisa Thölken, Julia Welzel

**Affiliations:** Klinik für Dermatologie und Allergologie, Universitätsklinikum Augsburg, Medizincampus Süd, Sauerbruchstr. 2, 86179 Augsburg, Deutschland

## Anamnese

**Abb. 1:**
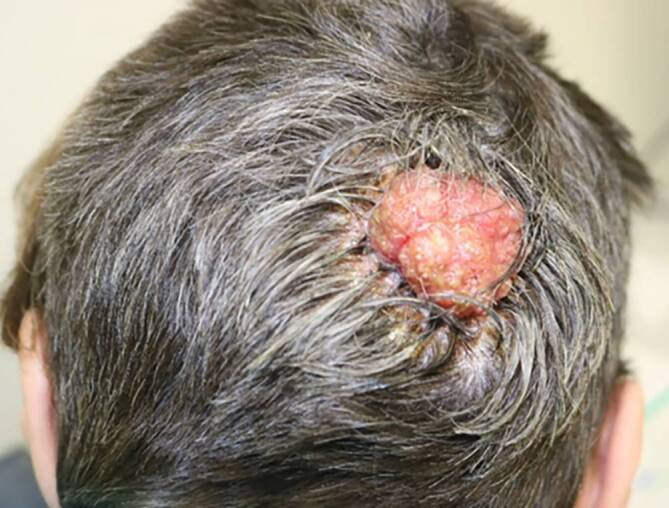
Okzipital rechts zeigt sich ein papillomatöser, rötlicher, flächig verkrusteter Tumor

Wir berichten über eine 63-jährige Patientin, welche sich notfallmäßig in unserer operativen Sprechstunde mit einem blumenkohlartig wachsenden Tumor am Hinterkopf vorstellte (Abb. 1). Bereits seit der Kindheit sei an der betroffenen Stelle eine kleine Hautveränderung vorhanden gewesen. In den letzten 3 Monaten sei diese Hautveränderung jedoch plötzlich schnell größenprogredient gewesen und habe angefangen zu nässen.

## Klinischer Befund

Klinisch zeigte sich okzipital rechts ein 4 × 4 × 3,4 cm messender, erythematöser, derber, schlecht verschieblicher und flächig verkrusteter Knoten (Abb. 1). Die Umgebung des Tumors zeigte sich ebenso wie das restliche Integument inspektorisch unauffällig.

## Diagnose und Therapie

**Abb. 2:**
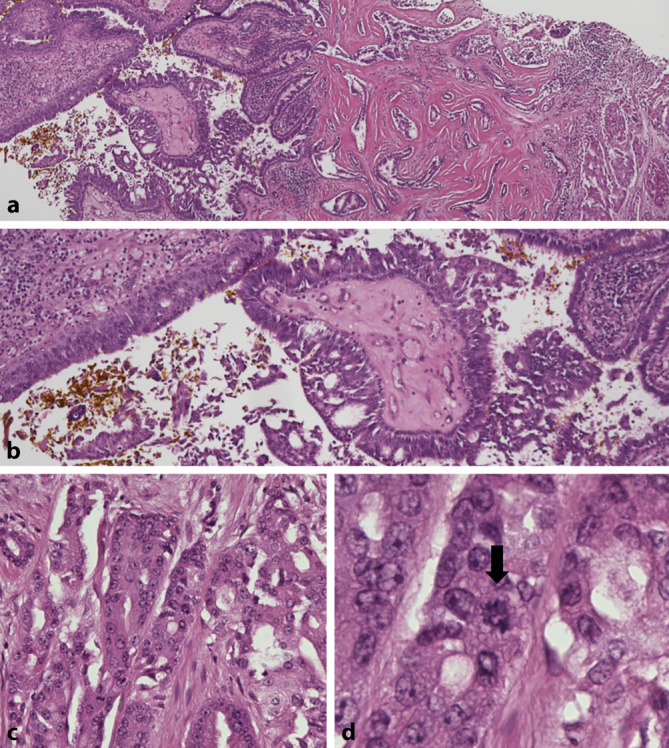
Histologisches Präparat gefärbt mit Hämatoxylin-Eosin. **a** Darstellung mehrerer Komponenten des Tumors mit Transitionszone des Syringocystadenoma papilliferum in ein In-situ-Karzinom sowie rechts im Bild Anteilen eines desmoplastischen Wachstums. **b** In-situ-Karzinom mit zellulären Atypien. **c** Polymorphismus und zelluläre Atypien. **d** Atypische Mitose markiert mit einem *schwarzen Pfeil*

**Abb. 3:**
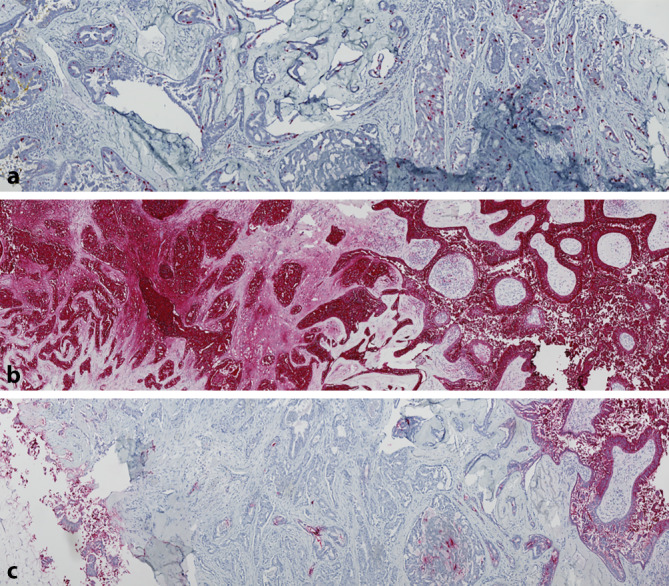
Immunhistochemische Färbungen. **a** Ki67, **b** EMA, **c** CEA

Bei initialem Verdacht auf ein lokal fortgeschrittenes Plattenepithelkarzinom wurde zunächst computertomographisch eine Infiltration der Kalotte ausgeschlossen. Anschließend wurde eine mikrographisch kontrollierte Exzision des Tumors im Rahmen einer mehrzeitig geplanten Operation in Lokalanästhesie durchgeführt. Der Wundverschluss erfolgte nach vollständiger Exzision mittels Doppelrotationslappenplastik. In der histologischen Untersuchung zeigte sich ein papillomatös wachsender, maligner Tumor, bestehend aus basaloid differenzierten Tumorzellen, welche sich teils strangartig im Korium anordnen und teils duktale Strukturen imitieren. Innerhalb des Tumors ließen sich mehrere Komponenten abgrenzen: Areale des Syringocystadenoma papilliferum (SCAP), eine Transitionszone mit In-situ-Karzinom sowie ein Bereich mit desmoplastischem Wachstum (Abb. 2a und b). Die Tumorzellen wiesen eine mäßige Zell- und Kernpleomorphie sowie atypische Mitosen auf (Abb. 2c und d). Peritumoral zeigte sich ein ausgeprägtes lymphozytäres Infiltrat. Squamöse Differenzierung oder Hinweise auf einen Naevus sebaceus waren nicht erkennbar. Immunhistochemisch wies der Tumor eine stark positive Reaktion mit EMA, CEA und Panzytokeratin bei mäßig erhöhter Proliferationsrate auf. In Anteilen des Tumors mit desmoplastischem Wachstum zeigte sich ein Verlust von CEA (Abb. 3). Für BerEp4 und Aktin zeigten sich die Tumorzellen negativ. Wir stellten den Verdacht auf ein Syringocystadenocarcinoma papilliferum.

Sonographisch ergab sich kein Anhalt für eine Lymphknotenmetastasierung. Da eine evidenzbasierte Nachsorgeempfehlung von Patienten mit Syringocystadenocarcinoma papilliferum nicht vorliegt, wurden zunächst halbjährliche klinische Untersuchungen angeraten.

## Diskussion

Das Syringocystadenocarcinoma papilliferum (SCACP) ist eine extrem seltene, maligne Neoplasie der Hautadnexe. Seit der Erstbeschreibung im Jahr 1980 sind bisher etwas mehr als 50 Fälle veröffentlicht worden. Es entwickelt sich meist ausgehend von einem Syringocystadenoma papilliferum (SCAP) und präsentiert sich als In-situ-Adenokarzinom und/oder invasives Adenokarzinom [1]. Etwa 40 % der Fälle treten in Assoziation mit einem Naevus sebaceus auf. Das SCACP entsteht typischerweise im Erwachsenenalter und zeigt keine geschlechtsspezifische Präferenz. Aufgrund der Seltenheit gibt es nur begrenzte Daten zur Inzidenz und Prävalenz dieser Entität [2, 3].

Makroskopisch kann sich das SCACP als Plaque oder Knoten mit hautfarbener bis rötlicher Färbung präsentieren. Über 50 % der beschriebenen Fälle betreffen die Kopf-Hals-Region [4]. Charakteristisch ist die auf dem Tumor aufliegende Kruste, die durch die Dekapitationssekretion der apokrinen Drüse verursacht wird [5]. Histologisch stellt sich das SCACP morphologisch in Analogie zum benignen Gegenpart, dem SCAP, als papillomatöser Tumor mit in die Dermis eingezogenen, erweiterten Drüsenformationen dar. Die Drüsenformationen weisen eine Zweischichtung, welche aus einem äußeren kuboidalen Myoepithel und einem inneren Zylinderepithel besteht, auf. Im Gegensatz zum SCAP zeichnet sich das SCACP jedoch durch das Vorhandensein von zytomorphologischen und architektonischen Malignitätskriterien wie Zell- und Kernpleomorphien, Vorliegen von atypischen Mitosen und einem tief und diffus infiltrierenden Wachstumsmuster aus [4]. Ein spezifisches immunhistochemisches Profil zur Differenzierung zwischen einem SCACP und einem nichtkutanen Adenokarzinom ist nicht bekannt. In dem hier beschriebenen Fall zeigten sich die Tumorzellen passend zur apokrinen Herkunft des Tumors deutlich positiv für EMA (epitheliales Membranantigen) und Panzytokeratin. Für CEA (carcinoembryonales Antigen) zeigte sich ein partieller Verlust insbesondere in Anteilen des Tumors mit desmoplastischem Wachstum (Abb. 3).

Die Diagnose wird häufig erst nach mehreren Jahren gestellt, da der Tumor wenig bekannt ist und klinisch mit zahlreichen anderen kutanen Neoplasien wie beispielsweise dem Basalzellkarzinom, Plattenepithelkarzinomen oder dem amelanotischen Melanom verwechselt werden kann. Histologische Differenzialdiagnosen sind neben dem gutartigen SCAP beispielsweise das Hidradenoma papilliferum oder Metastasen von nichtkutanen Adenokarzinomen [4].

Therapeutisch wurden die Tumoren in den bisher publizierten Fällen zumeist mit variablem Sicherheitsabstand, teilweise mikrographisch kontrolliert, exzidiert [4, 7]. Die Rate an postoperativen Lokalrezidiven ist mit 30–40 % sehr hoch [1]. Bei einigen Fällen wurde zudem zusätzlich eine Wächterlymphknotenbiopsie durchgeführt [7]. Lokal fortgeschrittenere Tumoren oder Fälle mit lokoregionärer Metastasierung wurden mittels Radiatio oder Chemotherapie erfolgreich behandelt [6]. Regionale Lymphknotenmetastasen sind in etwa 20 % und Fernmetastasen in etwa 6 % der Fälle beschrieben [4, 8]. Bisher sind noch keine Empfehlungen für Staginguntersuchungen oder Nachsorgeschemata für diese seltene Tumorentität etabliert.

## Fazit für die Praxis

- Das Syringocystadenocarcinoma papilliferum ist ein extrem seltenes kutanes Adnexkarzinom, welches meist in Assoziation zu einem Syringocystadenoma papilliferum entsteht.
- Makroskopisch tritt es häufig als Tumor im Kopf-Hals-Bereich auf, oft begleitet von einer charakteristischen Kruste durch Sekretion der apokrinen Drüsen.
- Histologisch kann es mit Hautmetastasen von anderen Adenokarzinomen verwechselt werden.
- Aufgrund des hohen Lokalrezidivrisikos wird therapeutisch eine vollständige Exzision mit weitem Sicherheitsabstand empfohlen. Lokoregionäre oder Fernmetastasierung treten in bis zu 25 % der Fälle auf.
